# Cardiovascular effects of sub-daily levels of ambient fine particles: a systematic review

**DOI:** 10.1186/1476-069X-9-26

**Published:** 2010-06-15

**Authors:** Omar Burgan, Audrey Smargiassi, Stéphane Perron, Tom Kosatsky

**Affiliations:** 1 Département de santé environnementale et santé au travail, Université de Montréal, Canada; 2 Institut National de Santé Publique du Québec (INSPQ), 1301 Sherbrooke Est, Montréal (Québec), H2L 1M3, Canada; 3 Direction de Santé Publique de l'Agence de la Santé et des Services Sociaux de Montréal, Canada; 4 British Columbia Center for Disease Control, Canada

## Abstract

**Background:**

While the effects of daily fine particulate exposure (PM) have been well reviewed, the epidemiological and physiological evidence of cardiovascular effects associated to sub-daily exposures has not. We performed a theoretical model-driven systematic non-meta-analytical literature review to document the association between PM sub-daily exposures (≤6 hours) and arrhythmia, ischemia and myocardial infarction (MI) as well as the likely mechanisms by which sub-daily PM exposures might induce these acute cardiovascular effects. This review was motivated by the assessment of the risk of exposure to elevated sub-daily levels of PM during fireworks displays.

**Methods:**

Medline and Elsevier's EMBase were consulted for the years 1996-2008. Search keywords covered potential cardiovascular effects, the pollutant of interest and the short duration of the exposure. Only epidemiological and experimental studies of adult humans (age > 18 yrs) published in English were reviewed. Information on design, population and PM exposure characteristics, and presence of an association with selected cardiovascular effects or physiological assessments was extracted from retrieved articles.

**Results:**

Of 231 articles identified, 49 were reviewed. Of these, 17 addressed the relationship between sub-daily exposures to PM and cardiovascular effects: five assessed ST-segment depression indicating ischemia, eight assessed arrhythmia or fibrillation and five considered MI. Epidemiologic studies suggest that exposure to sub-daily levels of PM is associated with MI and ischemic events in the elderly. Epidemiological studies of sub-daily exposures suggest a plausible biological mechanism involving the autonomic nervous system while experimental studies suggest that vasomotor dysfunction may also relate to the occurrence of MI and ischemic events.

**Conclusions:**

Future studies should clarify associations between cardiovascular effects of sub-daily PM exposure with PM size fraction and concurrent gaseous pollutant exposures. Experimental studies appear more promising for elucidating the physiological mechanisms, time courses and causes than epidemiological studies which employ central pollution monitors for measuring effects and for assessing their time course. Although further studies are needed to strengthen the evidence, given that exposure to sub-daily high levels of PM (for a few hours) is frequent and given the suggestive evidence that sub-daily PM exposures are associated with the occurrence of cardiovascular effects, we recommend that persons with cardiovascular diseases avoid such situations.

## Background

Exposure to higher than usual levels of ambient particulate air pollution over the course of several hours to several days has been shown to contribute to acute cardiovascular events such as arrhythmia, ischemia and myocardial infarction, which all could lead to emergency department visits, hospitalisation and death [1-3].

**Figure 1:**
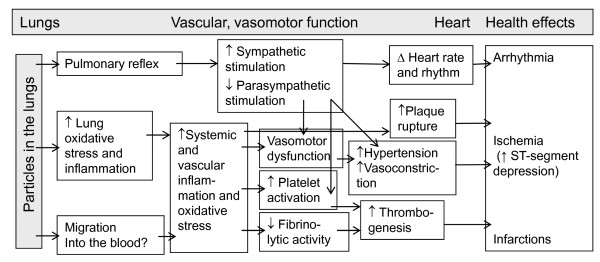
**Likely mechanisms whereby sub-daily exposures of fine particles (PM) induce acute cardiovascular effects (arrhythmia, ischemia and infarction)**. Adapted from Brook et al. [1] and Mills et al [3].

Various physiological pathways have been proposed to explain such cardiovascular effects. As presented in figure 1, likely mechanisms by which sub-daily levels of fine particles (PM) induce acute cardiovascular effects may involve the autonomic nervous system, systemic inflammation, vasomotor dysfunction and/or thrombogenesis [1-3].

While acute effects appear to occur soon after exposure to ambient PM [2], only recently have studies addressed whether cardiovascular events and exacerbations of respiratory disease symptoms associated with exposure to ambient PM relate mainly to brief (minutes or hourly) high-level exposure of particles or if they result from exposure averaged over an entire day. As such, sub-daily levels of PM are not regulated as is the case with other air pollutants for which sub-daily standards have been set (e.g. 1 h standard for ozone). Furthermore, guidance for vulnerable persons faced with high sub-daily exposure situations is lacking even if such exposures occur in many real-life situations such as in traffic jams, at bus stops, during accidental fires and explosions, in arenas, in indoor parking garages, during fireworks displays, etc.

Much of our understanding of the epidemiology of short-term ambient PM exposure derives from the linkage of administrative records of population health data with air pollution levels obtained from fixed site ambient monitors. Given that the precise timing of death, hospital admissions, and emergency room visits, are rarely available in administrative databases, epidemiological assessments based on such databases have not adequately addressed the effects of sub-daily high-level ambient particulate exposures.

We conducted a theoretical model-driven non-meta-analytic systematic literature review of published studies performed in humans, with the objective of providing a comprehensive evaluation of the cardiovascular effects of sub-daily exposures (≤6 hours) to ambient PM. A model-driven synthesis is a review that incorporates a conceptual model (see figure 1) and that attempts to provide information about the set of relationships among constructs of variables [4]. We chose this approach in an attempt to delineate information gaps meriting further research with regards to the likely mechanisms whereby sub-daily exposures to PM may induce acute cardiovascular effects. The model was used to define the keywords and, to organise and analyse the existing research.

The main questions addressed by this review are: 1) Does short duration exposure (sub-daily) to PM induce cardiovascular effects such as arrhythmia, ischemia and infarction?; 2) Do cardiovascular effects following short duration exposure to PM occur quickly after exposure?; 3) What are the likely mechanisms whereby sub-daily exposures to PM may induce acute cardiovascular effects such as arrhythmia, ischemia and infarction?

## Methods

### Data Sources

The bibliographic databases consulted were Medline and Elsevier's EMBase, using the Ovid portal. The temporal limits used were January 1996 to December 2008. Our search started in 1996 as no information was yet published on the subject before 1996, according to the 2004 U.S. Environmental Protection Agency Air quality criteria document for particulate matter [2].

### Data Extraction

The search strategy consisted of a combination of descriptors representing 1) cardiovascular effects (e.g. myocardial infarction), 2) the pollutant of interest (e.g. ambient PM) and, 3) the short duration of the exposure (e.g. short-term, peaks). The limits defined the age groups in which the study was performed (adults, age ≥ 18 years) and the language in which the study was published (English).

Only peer-reviewed scientific articles were retrieved. They were then selected by hand according to the following exclusion criteria: occupational studies; studies where exposure was to tobacco smoke, studies performed in animals, studies where there was no exposure to PM or studies where neither myocardial infarction (MI), arrhythmia, ischemia, nor vasomotor tone, thrombogenesis or heart rate and rhythm were measured. Commentaries, opinion and review articles were not analysed and neither were studies where exposure was for durations greater than 6 hours. Furthermore, only one publication of the same study/database was considered. The references cited in the selected articles were then searched to ensure completeness of the information gathered.

### Data Synthesis Method

Selected studies were organised into groups addressing specific sets of relationships among the construct of variables, presented in Figure 1. This figure was based on two important reviews by Brook et al. [1] and Mills et al. [3]. The following information was extracted from each article: design (human experimental or epidemiological studies), population characteristics (age and the presence of pre-existing cardiovascular disease; the latter were assumed to be on medication), particulate exposure characteristics (mass and number concentrations of particles with median diameters smaller than 10 μm, elemental and organic carbon levels and, duration and lags used) as well as presence of an association with the following health effects or physiological assessments: ST-segment depression, cardiac arrhythmia, MI, vasoconstriction assessed with blood pressure measurement, brachial artery diameter (BAD), flow mediated dilation (FMD) or forearm blood flow (FBF), changes in the standard deviation of all the normal-to-normal intervals (SDNN) on electrocardiogram and increased coagulation assessed with fibrinogen, platelet or tissue-plasminogen activator (t-PA) antigen levels. ST-segment depression (as a proxy for ischemia), arrhythmia and MI were considered as health effects; the other physiological assessments were analysed as measurements made to clarify mechanisms whereby sub-daily exposures to PM induce acute cardiovascular effects.

In the selected articles assessing the association between exposure to sub-daily levels of PM and heart rate variability measures, both time and frequency domains have been assessed but for simplification, here we only report on SDNN. Furthermore, blood markers of coagulation other than fibrinogen, platelets and t-PA were identified in the literature but as they were not consistently measured in many studies, they were not considered in our analysis. Moreover, associations with pollutants other than to particulate matter are not reported in the present study. When studies reported on the effects of both sub-daily and daily exposure to PM, we only reported the effects of sub-daily exposure as we did not review all studies where exposure to PM was longer than 6 hours in duration (including 24 hour studies) and only in a minority of studies that we reviewed were both hourly and daily exposure durations reported.

For our assessment of the literature, we defined a lag as the difference in hours between the end of the exposure and a physiological assessment or a health effect. For example, if MI occurred at 17 h00 and an exposure to PM at 14 h00 to 16 h00 is being considered, then the lag is 1 h and the duration of exposure is 2 h. In some instances, particularly in experimental studies, lag0 also included assessments made during the exposure. We report results for all lags assessed but again only results where exposure duration was 6 hours or less. We identified the presence of an association based on statistical significance. We did not perform a meta-analysis of available studies. A meta-analytic review that examines the magnitude of effects at different lags, for different outcome and PM size, is beyond the scope of this article. The objective of our comprehensive review was to assess whether acute effects of sub-daily PM exposures have been reported. The method used to quantitatively assess the evidence for each set of relationships was limited to vote-counting of statistically significant associations [5]. An association was considered 'suggestive' if more than half of the studies measuring the same effect showed an association in the same direction. If there were associations in fewer than half of the studies or, if the results were contradictory, we considered that there was limited evidence.

## Results

### Description of studies

**Figure 2:**
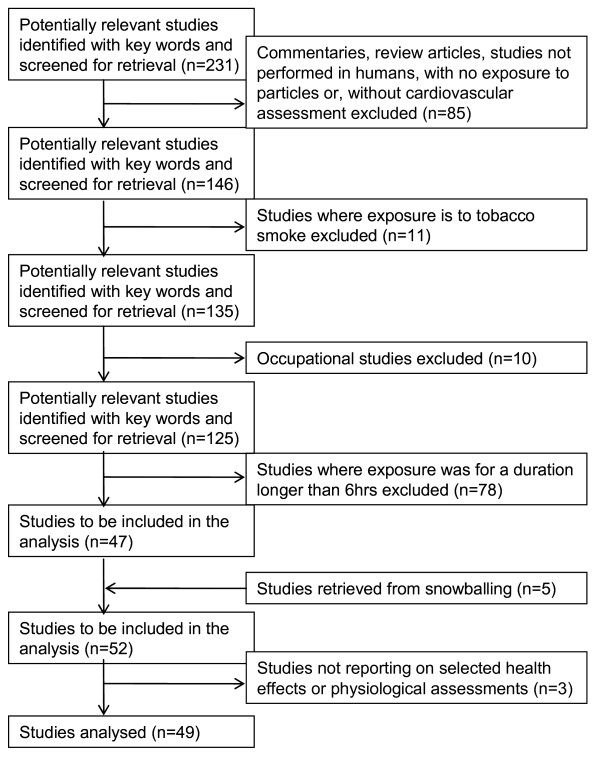
**Progress through the stages of the selection of studies for analysis**.

Figure 2 presents a flow chart of the stages of the selection of the studies for analysis. The initial key word search yielded 235 articles but four articles were excluded to retain only one article per study. Applying the above mentioned inclusion/exclusion criteria left 47 articles. To these 47 articles in which the duration of exposure was under or equal to 6 hours, five more articles were obtained from reference snowballing, for a total of 52 articles reviewed.

The information retrieved from 49 of the 52 studies is organised into four tables (Table 1, Table 2, Table 3 and Table 4) addressing the following sets of relationships with sub-daily exposures to PM that can be derived from Figure 1: i) cardiovascular effects (n = 17): five studies assessed ST-segment depression [6-10] which most likely indicated ischemia, eight studies arrhythmia or fibrillation [9,11-17] and, five studies were on MI [18-22]; ii) heart rate variability (SDNN): n = 20 [15-17,23-39]; iii) vasoconstriction: n = 17 [9,10,15-17,36,38,40-49]; iv) coagulation: n = 15 [9,10,15,17,36,38,39,43,44,48,50-54]. The remaining three studies [55-57] were excluded as they did not report on the presence of an association with the health effects or physiological assessments presented in Table 1, Table 2, Table 3 and Table 4 which were selected for analysis. While table 1 groups studies examining cardiovascular effects, Table 2, Table 3 and Table 4 group those addressing likely mechanisms by which sub-daily PM exposure cause cardiovascular effects.

**Table 1:** Evidence of association between sub-daily exposure to PM and arrhythmia, ischemia and myocardial infarction^a^

Authors	Subjects	Design	Exposure Levels	**Dur**.	Lags	Effect
**ST-Segment depression**						

Chuang et al., 2008	Aged 43-75 yrs, with CVD	Epi.	Fixed site PM2.5 mean daily levels: 50th: 9 μg/m3, (max 40); BC measured;	1 to 6 h^b^	Lag0	-

Gold et al., 2005	Aged 61-88 yrs	Epi.	Fixed site PM2.5, 12 h mean levels: 10^th^: 4, 90^th^: 26 μg/m3; BC also measured;	1 h^b^	Lag1 to 12 h	↑ at lag1 to 12 with BC

Lanki et al., 2008	Mean age 68 yrs (SD7), with CVD	Epi. Assessment during exercise	Fixed site PM2.5 hrly levels: 25^th^: 9, 75^th^: 18 μg/m3; fixed site UFP also measured; personal measurements made;	1 and 4 h^b^	Lag0 to 24 h;	↑ at lag1 to 10 with PM2.5 1 h; ↑ with 4 h dur. pre-testing

Gong et al., 2003	Aged 18-45 yrs, 50% asthmatics	Exp.	CAP~PM2.5 : 174 μg/m3 (SD37) with exercise;	2 h	Lag0_4^c ^4_22 h	-

Mills et al., 2007b	Mean age 60 yrs (SD1), with prior MI	Exp.	Diesel PM : 300 μg/m3 with exercise;	1 h	Lag0	↑ at lag0

**Fibrillation -arrhythmia**						

Ljungman et al., 2008	Mean age 62 yrs (SD13), with ICD	Epi.	Fixed site PM10 mean 2 h levels: 2 cities 50th: 19-15 μg/m3; PM2.5 measured in one city;	2 h^b^	Lag0	↑ v. arrhythmia with PM10; - with PM2.5

Rich et al., 2006	Aged 45-78 yrs, with ICD	Epi.	Fixed site PM2.5 hrly 50th: 9 μg/m3 (25^th^: 6, 75^th^: 15); BC also measured;	1 h^b^	Lag0	- a. fibrillation

Rich et al., 2006b	Mean age 63 yrs (20-88), with ICD	Epi.	Fixed site PM2.5 hrly 50th: 16 μg/m3 (25^th^: 12, 75^th^: 22); BC and OC also measured;	6 h	Lag0	- v. arrhythmia

Rich et al., 2005	Mean age 64 yrs (19-90), with ICD	Epi.	Fixed site PM2.5 hrly 50th: 9 μg/m3 (25^th^: 16, 75^th^: 15); BC also measured;	2 and 6 h^b^	Lag0	- v. arrhythmia

Gong et al., 2008	Aged 18-50 yrs, 45% asthmatics	Exp.	CAP~UFP : 100 μg/m3 (SD68) with exercise;	2 h	Lag0_4^c ^4_22 h	- ectopic beats

Gong et al., 2004	Mean age 36 yrs (SD11), 75% asthmatics	Exp.	CAP~PM10 : 157 μg/m3 (SD41) with exercise;	2 h	Lag0_4^c ^4_22 h	- ectopic beats

Gong et al., 2004b	Aged 54-85 yrs, 68% COPD	Exp.	CAP~PM2.5 : 167 (SD27) μg/m3; with exercise;	2 h	Lag0_4^c ^4_22 h	- ectopic beats^d^

Gong et al., 2003	Aged 18-45 yrs, 50% asthmatics	Exp.	CAP~PM2.5 : 174 μg/m3 (SD37) with exercise;	2 h	Lag0_4^c ^4_22 h	- ectopic beats

**Myocardial Infarction and cardiac arrest**						

Murakami et al., 2006	Deaths for MI, mean age 72 yrs (SD13)	Epi.	Fixed site PM7 hrly 50th: 44 μg/m3 (0-1093);	1 to 6h^b^	Lag0	↑

Peters et al., 2005	MI subjects, mean age 60 yrs (25-74)	Epi.	Fixed site PM2.5 hrly 50th: 15 μg/m3 (25^th^: 11, 75^th^: 20); UFP also measured; Exposure to traffic;	1 h^b^	Lag0 to 6 h and 24 h	- with PM2.5 or UFP; ↑ with traffic at lag0 to 6 h

Peters et al., 2001	MI subjects, mean age 62 yrs (SD13)	Epi.	Fixed site PM2.5 hrly mean: 12 μg/m3 (SD9);	1 h^b^	Lag0 to 5 h and 24 h	↑ at lag0 to 2 h

Rosenthal et al., 2008	Cardiac arrest subjects, mean age 61 yrs (SD17)	Epi.	Fixed site PM2.5 hrly 50th: 14 μg/m3 (10^th^: 6, 90^th^: 30);	1 and 4 h^b^	Lag0 to 8 h	↑ at lag0 and 1 h

Sullivan et al., 2005	MI subjects, mean age 69 yrs (21-98)	Epi.	Fixed site PM2.5 hrly 50th: 8.6 μg/m3 (2-147);	1, 2, 4 h^b^	Lag0	-

^a ^Numbers are rounded to the nearest integer for simplification; ^b ^Exposure durations longer than 6 h also studied; ^c ^Lag0_4 includes up to 90 minutes before the beginning of exposure; ^d ^Inconsistent results were reported between population subgroups (e.g. normal and asthmatics);

Abbreviations: a. fibrillation: Atrial fibrillation; BC: Black carbon; CAP: Concentrated ambient particles; CVD: Cardiovascular disease; COPD: Chronic obstructive pulmonary disease; Dur.: Duration of exposure; Epi.: Epidemiological; Exp.: Experimental; hrly: Hourly; ICD: Implantable cardioverter defibrillators; MI: Myocardial infarction; OC organic carbon; PM: Fine particles; SD: Standard deviation; UFP: Ultrafine particles; v. arrhythmia: Ventricular arrhythmia; -: No significant statistical association;

**Table 2:** Evidence of association between sub-daily exposure to PM and SDNN^a^

Authors	Subjects	Design	Exposure Levels	**Dur**.	Lags	SDNN
Adar et al., 2007	Aged 62-92 yrs, 82% HT	Epi.	PM2.5 mean on bus: 17 μg/m3 (SD10); BC also measured;	5', 30', 1, 4 h^b^	Lag0	↓ with PM2.5 and BC (more on bus (5' dur.)

Chang et al., 2007	Mean age 66 yrs (SD7), 47% CVD, 60% HT	Epi.	Mean personal PM2.5: 31 μg/m3 (SD27); Other PM size also measured;	1 to 6 h^b^	Lag0	↓ with PM2.5-10; ↑ with PM1-2.5

Chuang et al., 2007	Mean age 70 yrs (SD12), with or at risk of CVD	Epi.	Fixed site PM2.5 hrly mean: 52 μg/m3 (SD40); SO42- and OC also measured;	1 to 4 h	Lag0	↓ mostly with SO42-

Chuang et al. 2005	Mean age 68.1 yrs (SD3.6), 39% CVD, 61% HT	Epi.	Mean personal PM1-2.5: CVD:11 μg/m3 (SD9) and HT 13 (SD8);	1 to 4 h	Lag0	↓ with PM0.3-1; - with PM1-10

Gold et al., 2000	Mean age 73 yrs (53-87), 29% CVD, 57% HT	Epi.	Fixed site PM2.5, 4 h mean: 15 μg/m3 (3-49); PM10 also measured;	1 and 4 h^b^	Lag0	↓ with PM2.5

Lipsett et al., 2006	Mean age 71 yrs (SD6), with CVD	Epi.	Fixed site PM2.5 daily mean at 2 sites: 23 μg/m3 (6-90) and 14 (5-52); PM10 also measured;	2, 4, 6 h^b^	Lag0	↓ with PM10; - with PM2.5

Magari et al., 2002	Mean age 43 yrs (SD13)	Epi.	Daily mean personal PM2.5: 150 μg/m3 (SD292);	15'to 6 h^b^	Lag0	↓ up to 6 h dur.

Luttmann-Gibson et al., 2006	Mean age 71 yrs (54-90), 84% CVD	Epi.	Fixed site PM2.5 daily mean: 20 μg/m3 (25^th^: 12, 75^th^: 25); SO42- and elemental carbon (EC) also measured;	4 and 6 h^b^	Lag0	-

Sullivan et al., 2005b	Median age 77 yrs (57-87), 62% CVD	Epi.	Hrly mean outside home PM2.5: 11 μg/m3 (3-40);	1 and 4 h^b^	Lag0	-

Vallejo et al., 2006	Mean age 27 yrs (21-35)	Epi.	Median personal PM2.5: 74 μg/m3 (25^th^: 49, 75^th^: 111);	30' to 210'	Lag0	-

Wheeler et al., 2006	Aged 49-76 yrs, 46% CVD, 54% COPD	Epi.	Fixed site PM2.5, 4 h mean:: 18 μg/m3 (25^th^: 12, 75^th^: 22);	1 and 4 h^b^	Lag0	-

Beckett et al., 2005	Mean age 35 yrs (23-52)	Exp.	UFP and fine Zn PM: 500 μg/m3;	2 h	Lag0, 3, 6, 11, 23 h	-

Devlin et al., 2003	Mean age 67 yrs (SD1)	Exp.	CAP < PM2.5: 41 μg/m3 (SD9); with exercise;	2 h	Lag0 and 24 h	-

Frampton et al., 2004	Aged 18-40 yrs, 57% asthmatics	Exp.	UFP: 10 or 25 μg/m3; with exercise;	2 h	Lag0, 3.5, 15, 21, 45 h	-

Gong et al., 2008	Aged 18-50 yrs, 45% asthmatics	Exp.	CAP~UFP: 100 μg/m3 (SD68) for 2 h with exercise;	2 h	Lag0, 4, 22 h	-

Gong et al., 2004	Mean age 36 yrs (SD11), 75% asthmatics	Exp.	CAP~PM10 : 157 μg/m3 (SD41) with exercise;	2 h	Lag0, 4, 22 h^c^	-

Gong et al., 2004b	Aged 54-85 yrs, 68% COPD	Exp.	CAP~PM2.5 : 167 (SD27) μg/m3; with exercise;	2 h	Lag0, 4, 22 h	-

Peretz et al., 2008	Aged 24-48 yrs	Exp.	Diesel PM: 100 and 200 μg/m3 for 2 h;	2 h	Lag0, 1, 4, 20 h	-

Routledge et al., 2006	Aged 52-75 yrs, 50% CVD	Exp.	C UFP: 50 μg/m3;	1 h	Lag0 and 4 h	↑

Scharrer et al., 2007	Mean age 29 yrs (SD8)	Exp.	Welding fume PM 50th: 3500 μg/m3 (1000-25300);	1 h	Lag 5 h	-

^a ^Numbers are rounded to the nearest integer for simplification; ^b ^Exposure durations longer than 6 h also studied;

Abbreviations: BC: Black carbon; C: Carbon; CAP: Concentrated ambient particles; COPD: Chronic obstructive pulmonary disease; CVD: Cardiovascular disease; Dur.: Duration of exposure; Epi.: Epidemiological; Exp.: Experimental; hrly: Hourly; HT: Hypertensive; OC Organic carbon; PM: Fine particles; SD: Standard deviation; SDNN: Standard deviation of all the normal-to-normal intervals; UFP: Ultrafine particles -: No significant statistical association;

**Table 3:** Evidence of association between sub-daily exposure to PM and vasoconstriction^a^

Authors	Subjects	Design	Exposure Levels	**Dur**.	Lags	Vasocon- striction
Harrabi et al., 2006	Mean age 75 yrs (SD5), 46% HT, 6% MI	Epi.	Fixed site PM10 hrly mean: 20 μg/m3 (SD10); SO42- and OC also measured;	1 h^b^	Lag0, 3, 5, 24 h	↓SBP at lag3 and 5 h

Brook et al., 2002	Mean age 35 yrs (SD10)	Exp.	CAP~PM2.5: 153 μg/m3 (SD35) + O3;	2-2.5 h	Lag0	↓BAD; -BP and FMD

Dales et al., 2007	Aged 18-50 yrs	Exp.	PM2.5 bus stop 1: 40 μg/m3 (SD20); PM2.5 bus stop 2: 10 μg/m3 (SD10); PM1 also measured;	2 h	Lag0	↓FMD with PM2.5 not with PM1; -BP

Frampton et al., 2004	Aged 18-40 yrs, 57% asthmatics	Exp.	UFP: 10 or 25 μg/m3; with exercise;	2 h	Lag0, 3.5, 15, 21, 45 h	-BP

Gong et al., 2008	Aged 18-50 yrs, 45% asthmatics	Exp.	CAP~UFP: 100 μg/m3 (SD68) with exercise;	2 h	Lag0, 4, 22 h	-BP

Gong et al., 2004b	Aged 54-85 yrs, 68% COPD	Exp.	CAP~PM2.5 : 167 (SD27) μg/m3; with exercise;	2 h	Lag0, 4, 22 h	-BP

Gong et al., 2004	Mean age 36 yrs (SD11), 75% asthmatics	Exp.	CAP~PM10 : 157 μg/m3 (SD41) with exercise;	2 h	Lag0, 4, 22 h	-BP

Gong et al., 2003	Aged 18-45 yrs, 50% asthmatics	Exp.	CAP~PM2.5 : 174 μg/m3 (SD37) with exercise;	2 h	Lag0, 4, 22 h	-BP^c^

Mills et al., 2008	Mean age 54 yrs (SD2), 50% CVD	Exp.	CAP~PM2.5 : 190 μg/m3 (SD37); with exercise;	2 h	Lag6 to 8 h	-FBF with vasodil. or not; -BP

Mills et al., 2007b	Mean age 60 yrs (SD1), with prior MI	Exp.	Diesel PM : 300 μg/m3; with exercise;	1 h	Lag6 to 8 h	-FBF with vasodil. or not; -BP

Mills et al., 2005	Aged 20-38 yrs	Exp.	Diesel PM : 300 μg/m3; with exercise;	2 h	Lag2, 6, 24 h	↓FBF with vasodil.; -BP

Peretz et al., 2008b	Aged 20-48 yrs	Exp.	Diesel PM: 100 and 200 μg/m3;	2 h	Lag0 and 3 h	↓BAD; -FMD and BP

Routledge et al., 2006	Aged 52-75 yrs, 50% CVD	Exp.	C UFP: 50 μg/m3;	1 h	Lag0 and 4 h	-BP

Rundell et al., 2007	Mean age 21 yrs (SD2)	Exp.	Nb of PM1 near busy road: 144K/cm3 (SD59K); with exercise;	for 30'	Lag0	↓BAD and FMD

Shah et al., 2008	Aged 18-40 yrs	Exp.	C UFP: 50 μg/m3; with exercise;	2 h	Lag0, 3.5, 21, 45 h	↓FBF post ischemia at Lag3.5

Törnqvist et al., 2007	Mean age 26 yrs (18-38)	Exp.	Diesel PM: 300 μg/m3; with exercise;	1 h	Lag 24 h	↓FBF with vasodil. at lag24; -BP

Urch et al., 2005	Aged 18-50 yrs	Exp.	CAP~PM2.5: 147 μg/m3 (SD27) + O3;	2 h	Lag0	↑DBP

^a ^Numbers are rounded to the nearest integer for simplification; ^b ^Exposure durations longer than 6 h also studied; ^c ^Inconsistent results were reported between population subgroups (e.g. normal and asthmatics);

Abbreviations: BAD: Brachial artery diameter; BC: Black carbon; BP: Blood pressure; C: Carbon; CAP: Concentrated Ambient Particles; COPD: chronic obstructive pulmonary disease; DBP: diastolic BP; Dur.: Duration of exposure; Epi.: Epidemiological; Exp.: Experimental; FBF: forearm blood flow measured by plethysmography; FMD: Flow mediated diameter; HT: Hypertensive; hrly: Hourly; MI: Myocardial infarction; O3: Ozone; OC organic carbon; PM: Particulate matter; SD: Standard deviation; SBP: systolic BP; UFP: Ultrafine particles; vasodil.: Vasodilators; -: No significant statistical association;

**Table 4:** Evidence of association between sub-daily exposure to PM and coagulation^a^

Authors	Subjects	Design	Exposure Levels	**Dur**.	Lags	PL	F	**t-PA Ag**^c^
Rückerl et al., 2007	Aged 51-76 yrs, with CVD	Epi.	Fixed site PM2.5 daily mean: 20 μg/m3 (SD15); UFP nb, PM10 also measured;	6 h^b^	Lag0, 6, 12, 18 h	↓ with UFP lag 0 and 18 h		

Carlsten et al., 2007	Mean age 25 yrs (21-43)	Exp.	Diesel PM: 100 and 200 μg/m3	2 h	Lag3, 6, 22 h	-		

Frampton et al., 2004	Aged 18-40 yrs, 57% asthmatics	Exp.	UFP: 10 or 25 μg/m3; with exercise;	2 h	Lag0, 3.5, 15, 21, 45 h	-	-	

Ghio et al., 2000	Mean age 26 yrs (SD1)	Exp.	CAP~PM2.5: 23-311 μg/m3; with exercise;	2 h	Lag18 h	-	↑	

Gong et al., 2008	Aged 18-50 yrs, 45% asthmatics	Exp.	CAP~UFP: 100 μg/m3 (SD68); with exercise;	2 h	Lag0, 4, 22 h	-	-	-

Gong et al., 2004b	Aged 54-85 yrs, 68% COPD	Exp.	CAP~PM2.5 : 167 (SD27) μg/m3; with exercise;	2 h	Lag0, 4, 22 h	-	-	

Gong et al., 2003	Aged 18-45 yrs, 50% asthmatics	Exp.	CAP~PM2.5 : 174 μg/m3 (SD37); with exercise;	2 h	Lag0, 4, 22 h	-	-	

Lucking et al., 2008	Aged 21-44 yrs	Exp.	Diesel PM: 350 μg/m3;	2 h	Lag2 and 6 h	-		

Mills et al., 2008	Mean age 54 yrs (SD2), 50% CVD	Exp.	CAP~PM2.5 : 190 μg/m3 (SD37); with exercise;	2 h	Lag2, 6, 8 h	↑ at lag2		-

Mills et al., 2007b	Mean age 60 yrs (SD1), with prior MI	Exp.	Diesel PM: 300 μg/m3; with exercise;	1 h	Lag6, 24 h			↓ at lag6

Mills et al., 2005	Aged 20-38 yrs	Exp.	Diesel PM: 300 μg/m3; with exercise;	1 h	Lag2, 6, 24 h	-		↓ at lag6

Routledge et al., 2006	Aged 52-75 yrs, 50% CVD	Exp.	C UFP: 50 μg/m3;	1 h	Lag4 and 24 h	-	-	

Salvi et al. 1999	Aged 21-28 yrs	Exp.	Diesel PM: 300 μg/m3; with exercise;	1 h	Lag 6 h	↑		

Scharrer et al., 2007	Mean age 29 yrs (SD8)	Exp.	Welding fume PM 50th: 3500 μg/m3 (1000-25300);	1 h	Lag 5 h		-	

Törnqvist et al., 2007	Mean age 26 yrs (18-38)	Exp.	Diesel PM: 300 μg/m3; with exercise;	1 h	Lag 24 h	-		-

^a ^Numbers are rounded to the nearest integer for simplification; ^b ^Exposure durations longer than 6 h also studied; ^c ^Except in Gong et al., 2008, t-PA antigen was measured following infusion of vasodilators;

Abbreviations: Ag: Antigen; C: Carbon; CAP: Concentrated Ambient Particles; CVD: Cardiovascular disease; COPD: chronic obstructive pulmonary disease; Dur.: Duration of exposure; Epi.: Epidemiological; Exp.: Experimental; F: Fibrinogen; MI: Myocardial infarction; PL: platelets; nb: number; PM: Particulate matter; SD: Standard deviation; t-PA Ag: tissue plasminogen activator antigen; UFP: Ultrafine particles; -: No significant statistical association;

In total, 25 studies were epidemiological assessments; most of these studies used a longitudinal design. Levels of exposure to ambient particles in the epidemiological studies were in the range of those observed today in major American and European cities: The epidemiological studies usually reported hourly or daily mean or median PM2.5 levels (PM with median diameter < 2.5 μm) at fixed monitoring sites below 20 μg/m^3^. Exposure levels in the experimental studies typically ranged between 100 to 300 μg/m3 for one to two hours, which is a high daily maximum value in epidemiological studies performed in American and European cities. Particulate exposure characteristics varied between studies and included different particulate size fractions from fixed sites and personal measurements for epidemiological studies. Most experimental studies, on the other hand, used either concentrated ambient particles (CAP) or diesel exhaust to expose volunteers. Furthermore, exposure usually occurred with intermittent periods of exercise. Studies included both young and elderly people, healthy or with cardio-respiratory conditions and participants under medication.

### Cardiovascular effects of sub-daily exposures to fine particles

According to our assessment of studies presented in Table 1, there is suggestive electrographic evidence that sub-daily exposures to PM decrease the ST-segment which most likely indicates ischemia of the myocardium: of five studies (two experimental) that assessed ST-segment depression during exercise, three found increased depression during and shortly after exposure. The experimental study which did not observe any effects exposed subjects aged < 50 years with no preexisting cardiovascular disease [9]. There are insufficient studies published to date to assess the effects of lags and duration of exposure. However, the experimental positive study by Mills et al. [10] suggests that ischemia during exercise can occur quickly after the start of exposure and that reversal of ischemia may occur within less than an hour of continued exposure.

As for fibrillation or arrhythmia, there is limited epidemiological evidence of effects of sub-daily exposures to PM in studies assessing patients with implantable defibrillators (n = 4) (Table 1): only one of four studies reported a positive association with sub-daily levels of PM. Studies performed by Gong et al, did not show association between sub-daily levels of PM and ectopic beats.

There is suggestive evidence that sub-daily exposures to PM induce MI (Table 1): while there are a limited number of studies addressing the issue, positive associations were found in four out of five epidemiological studies performed in older adults (mean age around 60 years); however there is insufficient information to assess if effects occur shortly after exposure or if effects become more pronounced as elapsed time since exposure increases.

### Mechanisms whereby sub-daily exposures to fine particles may induce cardiovascular effects

The involvement of the autonomic nervous system has been suggested as a potential mechanism whereby sub-daily levels of PM may induce cardiovascular effects. In our assessment of studies that measured heart rate variability (presented in Table 2), discrepancies in results were noted between epidemiological (n = 11) and experimental studies (n = 9): while most epidemiological studies showed a decrease in SDNN, experimental studies did not report any associations. Contrary to epidemiological studies, most experimental studies did not assess the association in people with pre-existing cardiovascular disease. Furthermore, most studies assessed the effects of exposure at lag0 (the hour before the physiological assessment). As such, variations in SDNN appear to occur shortly after exposure; it is however impossible so far to assess whether variations in SDNN occur only shortly after exposure.

Regarding studies that assessed measurements of vasoconstriction (n = 15), which were mainly experimental in design, results were contradictory (Table 3): while blood pressure (BP) was mainly invariable, suggestive evidence of vasoconstriction was observed in studies where flow mediated dilation (FMD) or forearm blood flow (FBF) following vasodilator injection was measured. A positive association was found for short [46] as well as longer lags [48].

Finally, results of the 15 studies that assessed the effects of sub-daily levels of PM on fibrinolytic activity and coagulation were generally negative for change in the levels of platelets and fibrinogen (Table 4). Some discrepancies observed with levels of t-PA (a regulator of the degradation of intravascular fibrin) may be associated with the exposure conditions, as suggested by experimental studies performed by the same authors [10,43,44,48]. According to these studies, it seems that diesel exposure would influence fibrinolytic activity [10,44] but not concentrated ambient particles (CAP) [43]. Furthermore, this association would be observed at lag 6 h [10,44] and not at lag 24 h [48].

## Discussion

This literature assessment was performed to answer three main questions. First, do sub-daily exposures to PM induce cardiovascular effects such as arrhythmia, ischemia and MI? Our assessment shows that there is suggestive evidence that exposure to sub-daily levels of PM is associated with MI and ischemic events (mainly in the elderly and in individuals with prior cardiovascular diseases). There is limited evidence of an association between sub-daily levels of PM and arrhythmia or fibrillation; discrepancies observed may be due to methodological issues (see below). Our analysis was conducted without considering the effect of variable PM size. Further studies are needed to address the influence of PM size and composition on the acute cardiovascular effects of sub-daily exposure to PM as it may influence PM toxicity. Furthermore, meta-analytic reviews for each outcome should be performed to elucidate the magnitude of the observed associations.

Secondly, this review also addresses mechanistic issues. The delay with which cardiovascular effects appear following sub-daily exposure to PM was examined. Results of our analysis show that there is limited evidence suggesting that the cardiovascular effects of short duration exposure to PM occur quickly following exposure. However there is one experiment by Mills et al. [10] that demonstrates more pronounced myocardial ischemia during exercise which occurs minutes after the start of exposure. Further studies (experimental and epidemiological) are thus needed to confirm that cardiovascular effects of sub-daily exposure to PM may occur shortly, or with a short lag period, after the end of exposure. Furthermore, the reversal of ischemia observed in this study during exposure [10] suggests that in studies assessing the cardiovascular effects of PM, the timing of the health assessment should be carefully selected. Indeed, health effects may be missed due to the design of the study if the health assessment is not made at the right time (i.e. at the beginning of the exposure period).

Thirdly this review also addresses the physiological processes underlying whereby short exposures to PM, as opposed to longer averaging periods, may induce acute cardiovascular effects such as ischemia and myocardial infarction. According to the results of the studies reviewed, there is suggestive evidence from epidemiological studies that a plausible biological mechanism whereby sub-daily PM levels may induce effects involves the autonomic nervous system (as shown by studies on SDNN) even though experimental studies did not indicate this.

Discrepancies observed between epidemiological and experimental studies that measured SDNN may be due to differences in the number of repeated measures or to the fact that younger subjects without preexisting cardiovascular diseases were recruited in experimental studies. Experimental studies need to assess effects in those with pre-existing cardiovascular diseases to confirm associations observed in epidemiological studies between sub-daily PM exposure and SDNN. Similar associations with SDNN, as those reported here by epidemiological studies, have been observed in occupational studies [58-60]. Occupational studies that we identified assessing the cardiovascular effects of exposure to sub-daily PM (not reviewed here) have mainly examined its association with heart rate variability. The involvement of the autonomous nervous system in the effects of sub-daily PM exposures has also been suggested by studies on cardiac repolarisation (not reviewed here): an increase in QTC interval and a decrease in T-wave amplitude with exposure to sub-daily levels of PM have both been reported [55,57]. Nonetheless, further studies are needed to clarify the pathological implications, the prognostic value of a short-term decrease in heart rate variability (HRV) [61]. Furthermore, considerations are also needed to better understand if a decrease in HRV is an indicator of an alteration of the sympathetic tone. Further reviews should look at other HRV time and frequency domain parameters than SDNN.

Other physiological mechanisms, such as vasomotor dysfunction, are also likely implicated in the acute cardiovascular effects of sub-daily exposure to PM but discrepancies remain. For instance, associations between these exposures and FBF were found for both short lags (<4 h) and long lags (24 h) but no associations were found for lags of 6 to 8 h [10,43]. While vasomotor dysfunction does seem to be involved in the cardiac pathophysiology of PM exposure, so far it is unlikely to underlie effects such as ischemia, occurring during or immediately after exposure (e.g. [10]) because of differences in lags between exposure and onset of changes between the two. It is possible that vasomotor dysfunction may be involved in effects occurring hours after exposure, perhaps following the release of inflammatory mediators.

We performed a non-meta-analytical systematic literature review of studies that assessed a number of selected cardiovascular effects and physiological measures. Our literature review was framed by a theoretical model (presented in Figure 1) and, our quantitative assessment of the evidence was limited to counting studies presenting statistically significant associations. We recognize the limitations of our approach to identify the presence of an association which was based on statistical significance. We also recognize the limitations of our evaluation of the evidence of an effect as either 'suggestive' or 'limited', which was based on the number of studies with significant effects versus non-significant effects (i.e. suggestive if more than half of the studies showed a statistically significant association in the same direction). Our approach to assess the evidence may be biased by the tendency to publish positive results. Furthermore, issues of design and power may have limited our ability to report real associations. However, we consider our approach reasonable as most epidemiological studies reviewed used a longitudinal design with numerous repeated measure and thus, high power. Furthermore, while the present assessment does not allow the quantification of effects, our mechanistic framework does allow consideration of both human experimental and epidemiological studies in assessing the cardiovascular effects of exposure to sub-daily PM exposures and identifies research gaps. Future systematic reviews, mainly assessing the mechanistic basis of sudden cardiovascular effects, should consider including studies that assess physiological measures not included in this analysis, such as markers of systemic inflammation (e.g. C-reactive protein) and other indicators of vasoconstriction or vasoconstriction agonists (e.g. endothelin).

Based on our assessment of the literature, it appears that further studies are needed to confirm that sub-daily exposure to PM is associated with MI and ischemia as well as to address the influence of the size and the composition of the PM. Additionally, the literature reviewed does not provide any clear tendency as to the effects of different concentrations of exposure: epidemiological studies using linear models did not provide an exposure effect threshold. More work is needed to characterise the dose-response relationships. Studies should also address whether the observed effects of sub-daily exposures to PM are due to other concurrent air pollutants or to the combined action of a number of contaminants. Indeed while in the epidemiological studies we reviewed subjects were exposed to mixtures of ambient air pollutants, in a number of experimental studies showing associations exposure was often to either diesel exhaust (itself a pollutant mixture) or to CAP (concentrated ambient particles). Furthermore, exposure assessment in environmental epidemiological studies, as opposed to the highly controlled human experimental studies, can only be observational and as such is imperfect in assessing true subject exposures especially in cases where central pollution monitoring station data is used. Whether or not this limitation is more of concern in epidemiological studies with shorter sub-daily exposure assessment rather than longer averaged exposures is not clear. Another issue that deserves consideration is the influence of smoking on the health response to PM. In most studies so far, smokers were excluded or, the effect of smoking was adjusted statistically (data not shown).

The physiological mechanisms underlying the associations between sub-daily exposure to PM and MI or ischemia need to be clarified. For instance, experimental studies can be designed to better understand the time course of such effects. It is difficult to sort out the time course of effects with epidemiological studies as exposure has usually not been instantaneous. The influence of pre-existing cardiovascular diseases and medication use, on the effects of sub-daily exposure to PM, also need to be determined.

Finally, studies are needed to clarify if cardiovascular effects of sub-daily exposure to PM are captured by daily exposure. To date only daily levels of PM are subjected to regulation whereas sub-daily exposures of PM are not regulated. Information on this issue may be provided by time-series studies, in assessing the association between both daily mean PM levels and, hourly levels, with daily deaths or emergency room visits for cardiovascular effects. To our knowledge, very few studies (e.g. [56]) have assessed this so far, even with the limited effort that it would require.

## Conclusions

Future studies should clarify the points discussed above, specifically to which PM size fraction, composition and concurrent pollutants on sub-daily exposure, the cardiovascular effects are associated and by what mechanisms. It is questionable whether obtaining sufficiently precise information on these determinants is feasible with data from central pollution monitoring stations as opposed to personal monitors or an experimental study design. Although studies are needed to strengthen the evidence, our assessment of the literature on the effects of sub-daily exposure to PM leads to the conclusion that exposures of under 6 hours (including exposure as short as 30 to 60 minutes) may be associated with the occurrence of cardiovascular effects such as ischemia and MI possibly in the elderly and in individuals with prior cardiovascular diseases. Given that exposure to sub-daily high levels of PM (for a few hours) is frequent, we recommend that those with cardiovascular diseases avoid such situations.

## Abbreviations

BAD: Brachial artery diameter; BP: Blood pressure; CAP: Concentrated Ambient Particles; FBF: Forearm blood flow measured by plethysmography; FMD: Flow mediated diameter; HRV: Heart rate variability; MI: Myocardial infarction; PM: Fine particles; SDNN: Standard deviation of all the normal-to-normal intervals; t-PA Ag: Tissue plasminogen activator antigen;

## Competing interests

The authors declare that they have no competing interests.

## Authors' contributions

AS and TK conceived the study. All authors developed the literature search strategy. OB and AS conducted the literature search. OB and AS abstracted the included papers for the detailed tables prepared for this paper. SP and TK reviewed the included papers. OB and AS wrote the initial draft of the manuscript, and the other authors all contributed to its development, particularly SP to the theoretical framework. All authors reviewed and approved the final manuscript.
